# *Rhodotorula glutinis* as a living cell liposome to deliver polypeptide drugs *in vivo*

**DOI:** 10.1080/10717544.2018.1551439

**Published:** 2019-02-11

**Authors:** Zhengbin Fei, Shiyu Li, Jiajia Wang, Yuzhe Wang, Zhenyou Jiang, Wenhua Huang, Hanxiao Sun

**Affiliations:** a College of Pharmacy, Institute of Genomic Medicine, Jinan University, Guangzhou, China;; b Department of Anatomy, School of Basic Medical Sciences, Southern Medical University, Guangzhou, China;; c Departments of Microbiology and Immunology, Jinan University, Guangzhou, China

**Keywords:** *Rhodotorula glutinis*, living cell liposome, polypeptide drugs

## Abstract

The potential advantages of recombinant microbes as oral drug carriers for curing diseases have attracted much attention. The use of recombinant oil microbes as living cell liposomes to carry polypeptide drugs may be an ideal polypeptide oral drug delivery system. GM4-ΔTS was constructed by LFH-PCR from *Rhodotorula glutinis* GM4, which was screened and preserved in our laboratory, and then transferred into choline-phosphate cytidylyltransferase (CCT), which is a rate-limiting enzyme for lecithin synthesis. The results showed that the CCT gene was highly expressed in the GM4-ΔTS strain and could significantly increase fatty acid and lecithin contents in GM4-ΔTS-PGK1-CCT. Moreover, insulin, H22-LP, and α-MSH were successfully introduced into cells *in vitro*, and the strain no longer proliferated *in vivo*, for safe and controllable polypeptide drug delivery. *In vivo*, normal mice were intragastrically administered with recombinant strains carrying insulin and α-MSH, and different levels of polypeptide drugs were detected in serum and tissue, respectively. Then, recombinant strains carrying insulin were administered to type II diabetes mellitus mice. The results showed that the strains could effectively reduce blood glucose levels in mice, which indicated that the recombinant strains could carry insulin into the body, and the drug effect was remarkable. Therefore, recombinant GM4-ΔTS-PGK1-CCT strains were successfully used as living cell liposomes to carry insulin, H22-LP, and α-MSH peptides into the body for the first time; additionally, these strains have enhanced safety, controllability, and efficacy.

## 1. Introduction

Phospholipid bilaminar vesicular drug delivery systems based on liposomes have been extensively studied as possible carriers of polypeptide drugs because of their similarities to cell membranes, biocompatibility, and low toxicity (Tosato et al., 2018). Recombinant microbial cells have been reported to be very promising as oral drug carriers (Deshpande et al., 2016; Lang et al., 2017; Liu et al., 2018; Zhang et al., 2018). Previous studies have used *Saccharomyces cerevisiae* as a vaccine carrier (Liu et al., 2016), made yeast into microcapsules as natural liposome-encapsulated drugs (Salari et al., 2015), and used yeast as a natural bio-capsule to deliver unsaturated fatty acids orally (Watanabe et al., 2014). Generally, there are four main advantages for the use of recombinant microorganisms as an oral drug carrier. (i) Drugs are not easily deactivated by gastrointestinal acid or proteases and a similar efficacy to a normal gastrointestinal delivery system can be obtained at a low dose (Blanquet et al., 2001). (ii) Compared with injection, the patient’ compliance is significantly increased. (iii) It can assist in the biotransformation of drugs in administration through the digestive canal. (iv) The rapid growth of microorganisms, continuous production, and controllable production methods are helpful (Steidler et al., 2000). At the same time, liposome-encapsulation of peptide drugs is one of the key and difficult points in the field of liposome drug delivery. Polypeptide drugs are mainly encapsulated in liposomes, but liposomes are easily oxidatively decomposed during storage and transportation *in vitro*. Additionally, digestion and absorption *in vivo* are easily affected by acids and enzymes, causing leakage of encapsulated components, which greatly affects the biological function and application of liposome polypeptide drugs. Therefore, using recombinant oil microbes as a living cell liposome for delivery of drugs and as a lower molecular lipophilic drugs delivery system has unparalleled potential advantages. However, research on yeast at home and abroad is mainly focused on their surface modification or polypeptide secretion to deliver drugs (Padkina & Sambuk, 2018). Using intracellular lipid droplets from high-yielding oil yeast as a liposome-encapsulated polypeptide and a live-cell polypeptide liposome has not been reported.

*Rhodotorula glutinis*, a common oil-producing *Rhodotorula*, is widely found in nature and mammals (Rittmann, 2008), and recent studies have demonstrated that *R. glutinis* could be a nonpathogenic gene-delivery vehicle (Li et al., 2013). Compared with other oil-producing yeasts, *R. glutinis* has unique advantages; for example, it can use various carbon sources to produce oil, and the oil yield is equivalent to more than 50% of its own dry matter (Wang et al., 2009; Schneider et al., 2013). In *R. glutinis* cells, oil exists in the following two forms: (1) in the form of body lipids, and the content is constant, and (2) in the form of storage fats, and 95% of the oil in the microorganism usually exists in the intracellular form of triglycerides (TAGs) composed of polyunsaturated fatty acids (PUFAs), which further form lipid droplets; that is, the oil exists in the cytoplasm in the form of lipid droplets or fat particles (Papanikolaou et al., 2007). Among them, CCT is a key enzyme in the phospholipid biosynthesis pathway, that is dominated by PUFAs (Mallampalli et al., 1995). It can directly catalyze the synthesis of cytidine-5′-diphosphate choline (CDP-choline) through cytidine triphosphate (CTP) and choline phosphate (Friesen et al., 2001), thus promoting the synthesis and accumulation of phosphatidylcholine. This process must have a highly active CCT enzyme for a microorganism involved in CDP-choline biosynthesis; however, CCT enzyme activity is generally low in microbial cells such as *R. glutinis* (Tsukagoshi et al., 1987). Therefore, CCT has become a key rate-limiting enzyme in the process of lipid synthesis and accumulation.

*Rhodotorula glutinis* CCTCC M 2012203 GM4, which was screened and preserved in the laboratory, is a new strain of *Rhodotorula* with high-yielding oil that has a similar fatty acid composition to the fatty acid ratio required by human (Rittmann, 2008). This strain has been tested for toxicity and safety according to China’s New Resource Food Management Regulations, and the experimental results in the Supplementary Information prove that the strain is nontoxic and could not proliferate in special medium (not including TS) or *in vivo*. Therefore, in this study, to make the oil from the *R. glutinis* GM4 strain more suitable for a drug liposome, we used this new strain as a material to knock out thymidylate synthase (TS) and modify the strain with a CCT enzyme through genetic engineering to improve the lipid contents of the intracellular lipid droplets. This strain is advantageous for drugs such as insulin, H22-LP, and α-MSH with few side effects and remarkable curative effects for entering the body. The recombinant *R. glutinis* no longer proliferates *in vivo* due to its lack of TS, and carries the polypeptide drug for safe and controllable release. It can accelerate the absorption of the polypeptide drug *in vivo*, making it more similar to a synthetic medicinal liposome, thereby providing a new strategy for realizing the use of *R. glutinis* as a living cell liposome to deliver polypeptide drugs.

## 2. Materials and methods

### 2.1. Strains, plasmids, and animals

The *R. glutinis* GM4 strain was grown in YPD medium and the *Rhodotorula toruloides*, *Saccharomyces cerevisiae*, and *Escherichia coli* in this study were routinely cultured. *Rhodotorula glutinis* strain GM4 was screened and preserved by our laboratory and could produce high-yield lipids and unsaturated fatty acids (Rittmann, 2008). *Saccharomyces cerevisiae* 2.1445 was used in this study for CCT gene isolation and amplification was obtained from the China General Microbiological Culture Collection Center (Beijing, China). The *R. toruloides* was used in this study for TS gene cloning was preserved in our laboratory. The *E. coli* DH5α used in this study for expression vector extraction was preserved in our laboratory.

The expression vectors pPICZ-rD and pYES2-GFP were constructed and preserved in our laboratory, and the recombinant plasmid pPICZ-PGK1-CCT was purchased from Takara (Dalian, China).

Clean BALB/c mice, weighing 18–20 g and 4–5 weeks of age were purchased from the Experimental Animal Center, Sun Yat-sen University, and were used throughout this study in accordance with the policies and regulations for raising and using laboratory animals. All mice were fed with standard mice feed and water and were bred under specific pathogen-free (SPF) conditions.

### 2.2. Construction of TS-deficient strain

The TS-knockout component was constructed by long flanking homology region-PCR (LFH-PCR). The GFP gene on the pYES2-GFP plasmid was amplified according to the TS gene sequence from the NCBI database and the sequence of pYES2-GFP plasmid. The primers used are as follows, with the underlined parts being complementary to the GFP gene sequence.

Primer A: 5′-CTTTCCTGCGTCGTCTACCA-3′;

Primer B: 5′-AGTGCTTCAGCCGCTACCC TTCAAGTCTGTAACCCATTCC-3′fan;

Primer C: 5′-TCACCTTGATGCCGTTCTT GTCGTGGGTTGTATCGGTTTA-3′ shun;

Primer D: 5′-GCCTTCCCGTTCTATTTCTTC-3′.

The GFP gene was amplified by primer B and C, and *R. toruloides* TS gene homologous sequence was added at both ends. The *R. toruloides* chromosome was used as a template. The first PCR was aimed at cloning the yeast chromosome homologous sequence, and two pairs of primers were used for PCR amplification. The second PCR cloned the GFP gene, using the pYES2-GFP plasmid as a template, with primer A and primer D; 5 μL of the first round of amplification products was added to the system for PCR amplification. Purification was performed according to the Omega Agarose Gel Kit operating instructions. The *R. glutinis* competent cells were prepared according to the transformation of yeast by the lithium acetate method (Tang et al., 2004). The competent cells were cultured in YPD solid medium containing thymidylate (50 μg·mL^−1^). Upon growth, a large single colony was picked with a sterile toothpick and inoculated into YPD liquid medium containing thymidylate under 250 r·min^−1^ shaking culture at 30 °C. The yeast chromosome was extracted as a template for transformant verification and to inoculate the correct GM4-ΔTS strain on the YPD plate containing thymidine for passage. After expansion, colonies were randomly selected, and the yeast chromosome was extracted as a template to further verify the stability of the exogenous fragment in the knockout strain. The successfully constructed GM4-ΔTS strain was sent to Synbio Technologies (Suzhou, China) for sequencing.

### 2.3. Construction of integration vector expressing exogenous CCT

**Figure 1.**
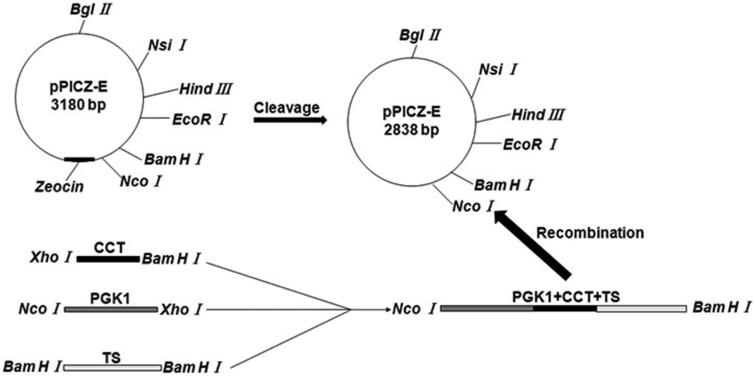
Construction of *Rhodotorula glutinis* integration vector pPICZ-PGK1-CCT-TS expressing exogenous CCT. *Escherichia coli* DH5α containing the pPICZ-rD plasmid was cultured and centrifuged to obtain the pPICZ-rD expression vector. To achieve efficient expression of the exogenous CCT gene under the strong promoter PGK1, we used overlap PCR to link the strong PGK1 gene and the CCT gene fragment and to ensure that no other sequences were present between the two gene fragments, thereby avoiding the abnormal expression of the target gene due to the introduction of other genes. The overlap PCR products and pPICZ-rD were digested with *NcoI* and *BamHI* respectively. After cleavage, the PGK1-CCT fragment and expression vector pPICZ-rD were purified, after which the purified PGK1-CCT fragment and pPICZ-rD expression vector were ligated using T4 DNA ligase to construct the pPICZ-PGK1-CCT recombinant plasmid.

The recombinant plasmid pPICZ-PGK1-CCT (Figure 1) was constructed by Takara (Dalian, China), and the recombinant plasmid was transformed into the GM4-ΔTS strain, which was named GM4-ΔTS-PGK1-CCT. Then, the positive clones were confirmed by PCR and double-enzyme digestion of the recombinant GM4-ΔTS-PGK1-CCT strain. PCR amplification was performed using the following specific primers: (1) CCT-F, 5′-ACTGAAGGGTCTGAGTTTGGG-3′ and (2) CCT-R, 5′- GAAGCGGAGGGAGTGATGG-3′.

### 2.4. Detection of plasmid stability during the passaging of the recombinant GM4-ΔTS-PGK1-CCT strain

The recombinant GM4-ΔTS-PGK1-CCT strain was inoculated into 30 mL of YPD liquid medium and cultured at 30 °C with shaking at 250 rpm. Then, 1 mL of the bacterial solution was diluted to 1:1 × 104 with sterile water and then coated on a common YPD plate; the total number of colonies and integrated vector colonies were calculated. A 1% inoculation sample was transferred to YPD liquid medium, cultured at 30 °C with shaking at 250 rpm. Twenty-four hours correlated with 10 generations, and this sample was cultured for 60 generations. Plate counts were performed every 10 generations. The formula for the plasmid stability is as follows:

$$\text{Stability }\left(\%\right)=\frac{\text{integrated vector colonies}}{\text{total colonies}}\times 100\%.$$

### 2.5. Determination of CCT expression levels in the recombinant GM4-ΔTS-PGK1-CCT strain

The correct recombinant plasmid was transferred to the GM4-ΔTS strain and then plated on a YPD plate that was cultured at 30 °C. After culturing for 20 h, the successfully constructed GM4-ΔTS-PGK1-CCT strain and GM4 strain were inoculated into 30 mL of liquid medium under 250 r·min^−1^ shaking culture at 30 °C. After culturing overnight, 1 mL of the bacterial solution was transferred to a new culture. After continuing to culture for 24 hours at 30 °C in the medium, 1.5 mL of the bacterial solution was removed and the thallus was collected by centrifugation (12,000 r·min^−1^, 2 min). The bacteria were resuspended with 100 μL of 1 × SDS loading buffer and then lysed and centrifuged at 8,000 r·min^−1^ for 10 min at room temperature to precipitate the cell fragments and DNA. Next, an appropriate amount of solution was applied for SDS-PAGE analysis. At the same time, the expression of the CCT enzyme in the recombinant GM4-ΔTS-PGK1-CCT strain was detected by Western blotting.

### 2.6. Analysis of the fatty acid and phospholipid compositions of the recombinant GM4-ΔTS-PGK1-CCT strain

The oil from the strain was extracted using the Soxhlet extraction method, and the oil content = the amount of oil from the bacteria/the biomass of the bacteria ×100%. The fatty acid composition of the recombinant GM4-ΔTS-PGK1-CCT strain was analyzed by gas chromatography (Fanali et al., 2017). The following GC conditions were used: GC-9870 gas chromatograph, the carrier gas was nitrogen, capillary column HP-5MS (30 m × 0.25 mm ×0.2 μm), flow rate of 1.0 mL·min^−1^, and a set temperature program. The program began at a starting temperature at 45 °C that was maintained for 1 min, after which the temperature was raised to 220 °C at a speed 4 °C·min^−1^ and then maintained for 30 min. At the time of injection, the temperature was 240 °C, the split ratio was 1:20, and the injection volume was 2 μL.

The total phospholipids in GM4-ΔTS-PGK1-CCT were extracted using the modified Bligh-Dyer method (Bligh & Dyer, 1959), and the phospholipid contents were measured by evaporative light-scattering detector (ESLD) analysis, wherein the mobile phase was methanol-chloroform-acetonitrile-water.

### 2.7. Detection of the position of the polypeptide in the cell by laser confocal microscopy

The recombinant GM4-ΔTS-PGK1-CCT strain competent cells were prepared with fifty micrograms of modified FITC-insulin, FITC-H22-LP, or FITC-α-MSH being dissolved and mixed with the competent cells. Exogenous insulin, H22-LP, and α-MSH peptides were introduced into the strain by electroporation. In total, 0.1 mL of GM4-ΔTS-PGK1-CCT-F insulin, GM4-ΔTS-PGK1-CCT-F H22-LP, and GM4-ΔTS-PGK1-CCT-F α-MSH strains were separately weighed in a 1.5 mL EP tube, centrifuged (3,000 rpm, 5 min), resuspended in distilled water, added with Nile Red dye, stored in the dark for 2–3 min, and then observed by confocal microscopy (TCS SP2). The green fluorescent laser source was at 488 nm and red was at 514 nm.

### 2.8. Quantitative analysis by fluorescence spectrophotometry

A concentration gradient FITC-insulin, FITC-H22-LP, or FITC-α-MSH standard solutions was separately prepared to measure the fluorescence intensity (Fs). Similarly, the fluorescence intensities F_x_ of GM4-ΔTS-PGK1-CCT-F insulin, GM4-ΔTS-PGK1-CCT-F H22-LP, and GM4-ΔTS-PGK1-CCT-F α-MSH solution were determined. The F_0_ value of the blank solution was determined in a 1 mL cuvette. The standard curve was prepared; the fluorescence intensities of insulin, H22-LP, and α-MSH in living cells were quantitatively analyzed using the excitation wavelength at 514 nm and the emission wavelength at 492 nm; the wild GM4 strain served as a control. The sample contents were calculated according to the standard curve.

Then, based on the amino acid sequence of the exogenous polypeptide, we used the Protparam software at http://www.expasy.org to predict the theoretical pI, instability index, aliphatic index, grand average of hydropathicity (GRAVY), and half-life of the peptide. The http://www.expasy.org ProtScale software was used to predict the hydrophobicity of the proteins, and curves were drawn to verify the hydrophobicity. The zero point was used as the boundary, and the more points above zero, the stronger the hydrophobicity and vice versa.

### 2.9. Preparation of freeze-dried powder of the recombinant strain

The recombinant insulin, H22-LP and α-MSH strains were respectively made in bacterial suspension. Then, a 1:1 × 10^9^ bacterial suspension per mL was prepared using the blood cell counting plate method before a lyophilized powder was prepared. Then, a certain amount of lyophilized powder was resuspended in the culture medium and inoculated onto a red yeast agar medium to further observe the growth state of the strain.

### 2.10. In vitro release of recombinant strain carrying polypeptide

A certain amount of lyophilized powder was weighed and dispersed in a PBS buffer solution at pH =7.4. The solution was transferred to a dialysis bag and immersed in a PBS solution at 37 °C to simulate *in vitro* release. At intervals, 1 mL of the sustained-release solution was removed and the same amount of fresh PBS buffer was added to keep the volume of the buffer constant. The absorbance of the sustained-release solution was measured by UV spectrophotometry. The cumulative release amount of the exogenous polypeptide from the recombinant strain was calculated according to the standard curve equation, and the curves for cumulative release rate and time of exogenous polypeptides in the *in vitro* recombinant strains were plotted.

### 2.11. Content and tissue distribution of insulin and α-MSH in serum from healthy mice

The mice were divided into 8 groups with six normal mice per group. The groupings were as follows: GM4-insulin lysate, GM4-ΔTS-PGK1-CCT-insulin, insulin peptide solution, GM4-α-MSH lyophilized powder solution, GM4-ΔTS-PGK1-CCT-α-MSH lyophilized powder solution, α-MSH lyophilized powder solution, and the control group, which was intragastrically administered normal saline. All the mice groups are fed the same dose. Five days after continuous administration, the serum insulin and α-MSH concentrations were measured with an ELISA kit with blood collection from the posterior eyeball venous plexus. Moreover, for insulin groups, the mice were sacrificed at 1, 2, 4, 8, 12, and 24 h after the gavage and the liver, heart, spleen, lung, and kidney were dissected. For the α-MSH groups, after the blood was collected from the eyeball, the mice were sacrificed immediately, and the liver, spleen, lung, and kidney were removed. Then, the appropriate tissue was cut out to make a homogenate and shaken at 200 rpm for 20 min at 25 °C. The supernatant was filtered with a 0.22 μm microporous membrane to remove impurities, and the insulin, and α-MSH concentrations were measured by ELISA kit.

### 2.12. Effect of the recombinant strain on blood glucose in mice with type II diabetes mellitus

A mouse model of type II diabetes was established by high-fat feeding and intraperitoneal injection of an alloxan solution. Successfully modeled diabetic mice were weighed and randomly divided into 5 groups. The insulin-administered group was administered insulin-containing PBS solution (50 U·kg^−1^, ig), the GM4-ΔTS-PGK1-CCT-insulin group with 1 × 10^10^ CFU GM4-ΔTS-PGK1-CCT-insulin bacterial solution, the insulin injection group was subcutaneously injected with insulin-containing PBS solution (50 U·kg^−1^, sc), the GM4-insulin group was administered GM4-carrying insulin solution (50 U·kg^−1^, ig), and the control group was given the same amount of normal saline. Then, the blood was taken from the caudal vein at 0, 1, 2, 4, 6, 9, 12 h after administration. After blood coagulation, centrifuged it at a low temperature. Then, accurately measured 20 μL serum, and blood glucose levels were measured by GOP-POD method. 

### 2.13. Statistical analysis

Statistical analysis was performed using GraphPad Prism 5.0 and Origin 8.0. All data in the figures and text are presented as the arithmetic mean ± standard deviation (SD). Data were representative of three or more independent experiments. The significance of differences among the groups was calculated with one-way ANOVA. In all cases, *p* < 0.05 was considered statistically significant.

## 3. Results

### 3.1. Validation of exogenous gene fragments and yeast transformants constructed by LFH-PCR

**Figure 2.**
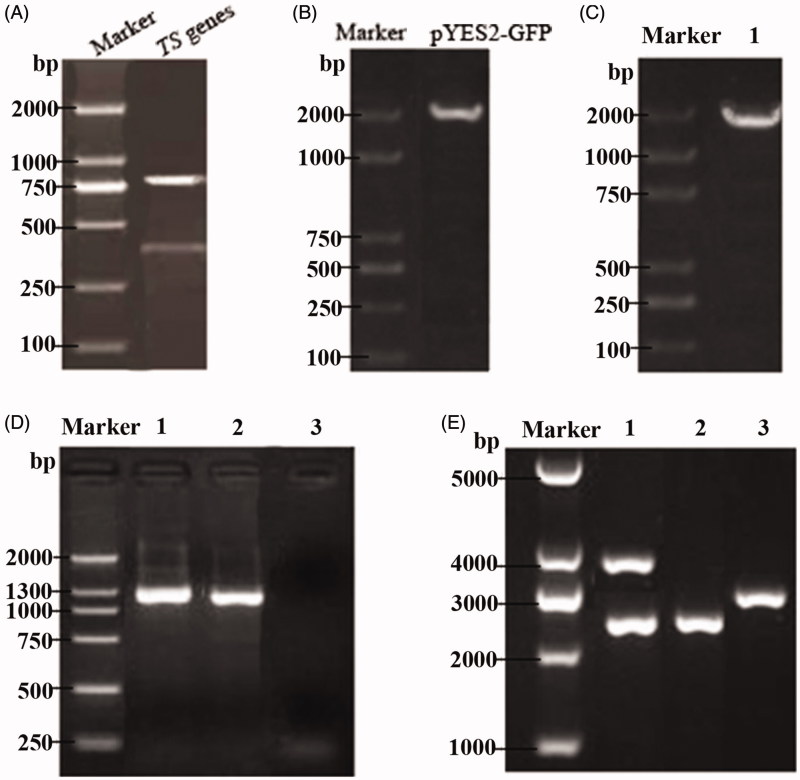
Identification of a thymidine synthase-deficient strain and its integrated pPICZ-PGK1-CCT-TS vector. (A) Agarose gel electrophoresis pattern of the upstream and downstream homologous sequences from the amplified TS gene, wherein the two bands shown are the two PCR products and represent the upstream and downstream homologous sequences of the TS gene. (B) Cloning electrophoresis identification of the GFP gene and its homologous sequences, with a size of approximately 1900 bp. (C) PCR identification of the TS gene knockout strain. Lane 1 is the GM4 strain with TS gene knockout. (D) PCR detection of the recombinant GM4-ΔTS-PGK1-CCT strain inserts CCT. Lanes 1 and 2 are the recombinant strains and lane 3 is an empty vector strain. (E) Identification of the pPICZ-PGK1-CCT-TS integration vector by double digestion. Lane 1 is the recombinant plasmid pPICZ-PGK1-CCT-TS. The results show that the upper band is an empty plasmid and a TS gene fragment, and the lower band is CCT gene fragment and the PGK1 promoter. Lane 2 is the pPICZ-rD plasmid from which the resistance gene was removed and lane 3 was pPGK1Z-rD (pPGK1Z-rD as a negative control).

The genomic DNA from *R. toruloides* was extracted as a template. Then, the upstream and downstream homologous sequences of the TS gene amplified by primers were analyzed by agarose gel electrophoresis to identify the two PCR products, and the results were consistent with the theoretical predictions from Figure 2(A) (top: 766 bp, bottom: 408 bp). The pYES2-GFP plasmid DNA was used as a template and A and D were used as primers. At the same time, 5 μL of the above PCR product was added to the reaction system for PCR amplification. The resulting amplified product was the GFP gene with the first amplification product sequence at both ends, with a size of 1981 bp by electrophoresis (Figure 2(B)), which was consistent with the theoretical prediction size.

The transformants were transferred into a competent GM4 strain; the yeast chromosome was extracted after culture and identified by the colony PCR. A and D were used as primers for amplification, and the amplified products were analyzed by gel electrophoresis. The product size was consistent with the above results (Figure 2(C)). To verify the stability of the transformants, the correct GM4-ΔTS colony was expanded and the chromosomes were extracted for PCR. The experimental results showed that the exogenous GFP gene could still be amplified after multiple passages, which indicated that the GFP gene had been integrated into the GM4 chromosome; thus, the strain was a TS gene knockout strain. The GM4-ΔTS strain was sent to Synbio Technologies (Suzhou, China), and its chromosomes were sequenced. The results showed that the GFP gene had been integrated and replaced the original TS gene.

### 3.2. Identification of the integration vector pPICZ- PGK1-CCT-TS

The recombinant pPICZ-PGK1-CCT-TS plasmid was constructed by Takara (Dalian, China), and the recombinant expression vector was electrotransformed into the competent GM4-ΔTS strain. PCR detection showed that an ∼1300 bp gene was obtained in lanes 1 and 2, which was consistent with the size of the CCT gene reported in GenBank, while the empty vector strain showed no bands (Figure 2(D)). The recombinant pPICZ-PGK1-CCT-TS plasmid was successfully transformed into the GM4-ΔTS strain, and the obtained recombinant strain was named GM4-ΔTS-PGK1-CCT. After GM4-ΔTS-PGK1-CCT was cultured, the plasmid was extracted and imaged after *Nco*I and *Bam*HI double digestion electrophoresis. Their bands were at ∼2500 bp and 4000 bp in lane 1 (Figure 2(E)). The fragment sizes after digestion were consistent with the inserted target gene fragment size, indicating that the target gene was inserted in the correct direction in the recombinant plasmid.

### 3.3. Expression of CCT in recombinant strain GM4-ΔTS-PGK1-CCT

**Figure 3.**
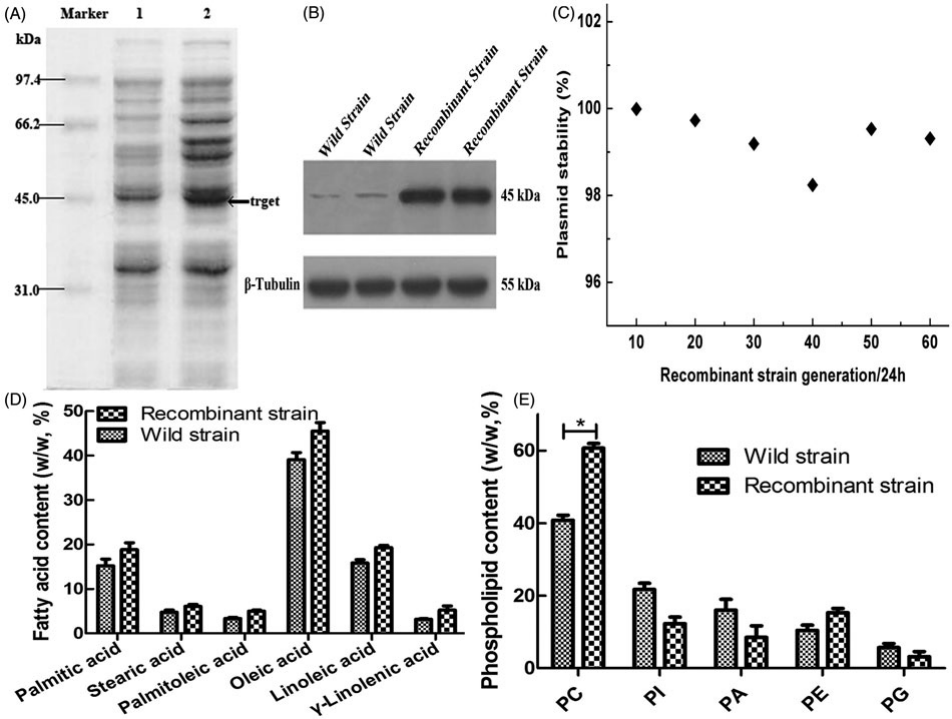
Expression of the CCT gene in the strain and its related effects. (A) Analyzed expression of the CCT gene in the strain by SDS-PAGE. Lane 1 is the recombinant GM4-ΔTS-PGK1-CCT strain, and lane 2 is the wild-type GM4 strain. (B) The expression of CCT gene in the strain was analyzed by Western blotting. β-Tubulin was used as the internal reference. Anti-PCYT1A was fixed and scanned after incubation. (C) The stability of the pPICZ-PGK1-CCT-TS plasmid in the transformed strain. (D) Analysis of the fatty acid composition of the recombinant and wild-type strains by GC. (E) Analysis of the phospholipid composition of the recombinant and wild-type strains by ESLD for phosphatidylcholine (PC), phosphatidylinositol (PI), phosphatidic acid (PA), phosphatidylethanolamine (PE), phosphatidylglycerol (PG). *p ˂ .05 compared to the wild-type strains.

The recombinant CM4-ΔTS-PGK1-CCT strain examined was performed by SDS-PAGE analysis with the GM4 strain being used as a control. The electrophoresis results (Figure 3(A)) showed an expression band at ∼45 kDa, which was consistent with the theoretical molecular mass of the CCT enzyme and was approximately twice as high compared to the wild-type strain. Western blotting with an anti-PCYT1A antibody revealed an ∼45 kDa protein from the recombinant strain CM4-TS-PGK1-CCT and GM4 strain (Figure 3(B)), which suggested that the CCT enzyme was expressed in the recombinant strains. Thus, the CCT enzyme is highly expressed in the recombinant strains.

### 3.4. Recombinant plasmid stability assay

The results from passages 20, 40, and 60 showed that the plasmid remained 99.3% stable after the passage 60th generation passage with the expressed bacteria (Figure 3(C)), indicating that the plasmid has good stability during passaging and meets the requirements for genetic engineering bacteria.

### 3.5. CCT promotes the accumulation of fatty acids and lecithin

The results of fatty acid analysis showed that the fatty acid contents in the recombinant strain were higher than in the wild-type strain (Figure 3(D)), indicating that transformation of the CCT enzyme could significantly improve the synthesis and accumulation of unsaturated fatty acids in the recombinant GM4-ΔTS-PGK1-CCT strain.

To study the effects of the CCT gene transfer on phospholipids in the GM4-ΔTS strain, the phospholipid contents were determined and analyzed during the experiment. We found that the phosphatidylcholine contents in the transformed strain increased significantly from 40.8% to 60.7% (Figure 3(E)). Thus, the transformed strain can accumulate more lecithin than the wild-type strain. Therefore, high CCT levels allow lecithin synthesis to be maintained at a high level.

### 3.6. LSCM to verify the location of the peptides into the cell

**Figure 4.**
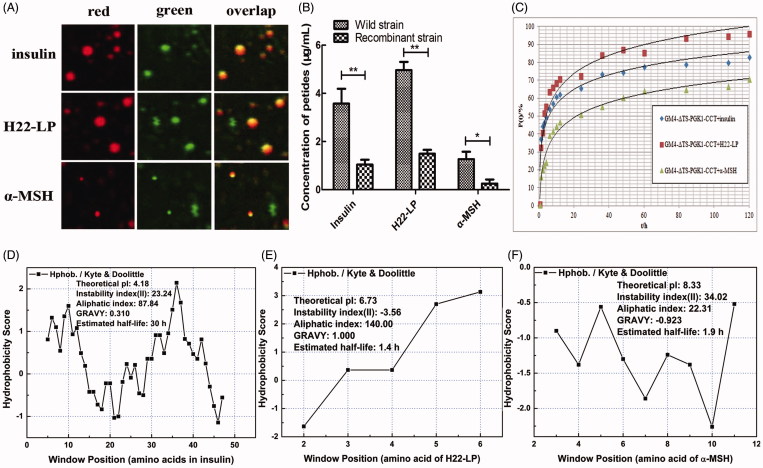
Effects of the exogenous polypeptide drugs transfected into the recombinant strains. (A) Analysis of the location of FITC-F-insulin, FITC-F-H22-LP, and FITC-F-α-MSH by CLSM and staining the lipid droplets with the Nile Red dye. The red fluorescent channel, green fluorescent channel, and an overlap of the red and green fluorescent images are shown. (B) The polypeptide contents of the exogenous polypeptide in the cell by electrotransformation. *p ˂ .05 and **p ˂ .01 compared with the wild-type strains. (C) The relationship between drug release rate P(t) and the time when the recombinant strain carried different exogenous polypeptides in PBS buffer. (D, E, and F) are based on the amino acid sequence of the polypeptide. The ProtScale software was used to predict the hydrophobicity of insulin, H22-LP, and α-MSH, and then the Protparam software was used to predict the theoretical pI, instability index, aliphatic index, and GRAVY of the proteins for further verification.

To detect the location of the exogenous polypeptides in the intracellular region of the recombinant strain GM4-ΔTS-PGK1-CCT, the lipid droplets were stained by Nile Red dye, which appeared red in the red fluorescent channel; because the polypeptide itself was labeled by FITC, it appeared green in the green fluorescent channel (Figure 4(A)). When we overlapped the two images, we observed that the red and green fluorescence spots coincided, and the overlapping color was yellow, indicating that the two substances exist in the same location. These results suggest that the three polypeptide drugs entered the cell and lipid droplets in the recombinant strain not only through lipophilic action, but also through other mechanisms.

### 3.7. Determination of intracellular polypeptides by fluorescence spectrophotometry

After electrotransformation, the bacterial liquid was collected by centrifugation to remove the polypeptide from the solution and the intracellular polypeptide contents were determined by fluorescence spectrophotometry. The experimental results are shown in Figure 4(B); the polypeptide drug contents entering the intracellular recombinant strain were significantly higher than in the wild-type strain, even for α-MSH, which has relatively weak liposolubility (Figure 4(C,D,E)). The recombinant strain intracellular contents of insulin, H22-LP, and α-MSH were detected as 3.58, 4.97, and 1.27 μg·mL^−1^, respectively. In contrast, the initial concentration of polypeptide drug was ∼10 μg·mL^−1^ at the beginning of electrotransformation, which indicated that the concentration of intracellular polypeptide drug is greater than 1/10 of the initial concentration, and with H22-LP, which has high fat-solubility, reaching up to ½ of the initial concentration.

From Figure 4(D,E,F), insulin, H22-LP, and α-MSH had higher aliphatic indexes (H22-LP > insulin > α-MSH), all of which belong to liposoluble proteins; thus, the polypeptide drugs enter the strains mainly because of the peptides’ fat-solubility. At the same time, the GRAVY of insulin, H22-LP, and α-MSH was 0.310, 1.000, and −0.923, respectively. The negative value indicates that the protein is hydrophilic, and the positive value is indicative of hydrophobic, demonstrating that insulin and H22-LP display hydrophobicity and α-MSH shows hydrophilicity. Thus, another reason for the entry of the polypeptide drugs into the strain is similarity and intermiscibility between the polypeptide drugs and the cell membrane component (Bhattacharya & Haldar, 2000).

### 3.8. In vitro release characteristics of exogenous polypeptides in recombinant strains

**Figure 5.**
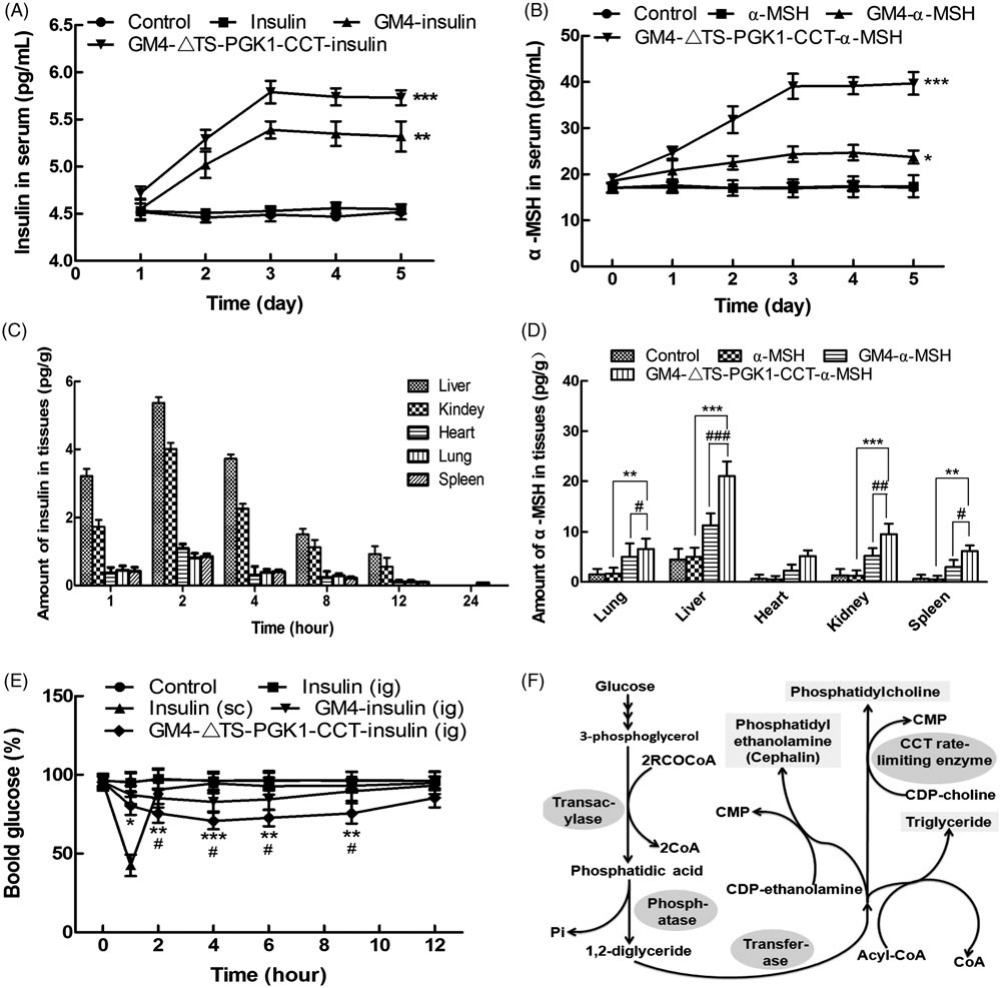
Pharmacological effects of the recombinant strains carrying insulin and α-MSH polypeptide drugs into mice. (A) Changes in the serum insulin concentrations with time in normal mice after oral administration of different drugs. *p ˂ .05 and ***p ˂ .001 compared to insulin. (B) Changes in the serum α-MSH concentration with time in normal mice after oral administration of different drugs. **p ˂ .01 and ***p ˂ .001 compared to α-MSH; ^###^*p* ˂ .001 compared to GM4-α-MSH. (C) The distribution of insulin in tissues in normal mice after 5 days of continuous gavage with different drugs. (D) Distribution of insulin in tissues in normal mice after 5 days of continuous gavage different drugs. **p ˂ .01 and ***p ˂ .001 compared to α-MSH; ^#^*p* ˂ .05, ^##^*p* ˂ .01, and ^###^*p* ˂ .001 compared to GM4-α-MSH. (E) Changes in the blood glucose with time in type 2 diabetic mice after intragastric administration of different drugs. *p ˂ .05 and **p ˂ .01 compared to insulin (ig); ^#^*p* ˂ .05, as compared to GM4-insulin (ig). (F) The glycerophospholipids biosynthetic pathway, in which CCTase acts as the rate-limiting enzyme in lecithin synthesis, and plays a key role in the phospholipid synthesis pathway.

To determine the drug-loading characteristics of the prepared recombinant strains carrying the exogenous polypeptides, we prepared a recombinant dried lyophilized powder carrying the exogenous polypeptide and then inoculated medium with the powder for 72 h. We observed that the strain can grow normally in TS-containing medium, but the survival rate in medium without TS was extremely low, ∼1%. Therefore, we further investigated the drug release rate P(t) of the recombinant strains carrying insulin, H22-LP, and α-MSH in PBS buffer at 37 °C and pH =7.4 as a function of time (Figure 5(C)). The recombinant strains carrying different exogenous polypeptides have different *in vitro* release characteristics. Approximately 32% of fat-soluble insulin and H22-LP is releases at 1 h, while α-MSH, which has a relatively low-fat solubility, was only 15.96% released, which was similar to the intracellular contents in the peptide-introduced strain. Moreover, the *in vitro* release characteristics of the three strains showed that the insulin, H22-LP, and α-MSH polypeptides could be released slowly after packaging of the recombinant strain and maintained for at least 5 days, indicating that the recombinant strain carrying the exogenous polypeptide has safe and controllable effects.

### 3.9. Study on the GM4-ΔTS-PGK1-CCT strain carrying insulin in healthy mice

To detect the ability of the recombinant strain GM4-ΔTS-PGK1-CCT to deliver insulin and α-MSH, blood was sampled from the eyeball after 5 days of gastric perfusion. The concentration of insulin in the serum was detected by ELISA kit; mice treated with the same volume of normal saline were used as a control group. As shown in Figure 5(A), in the GM4-insulin and GM4-ΔTS-PGK1-CCT-insulin groups, the serum insulin contents increased in 1–2 days and almost linearly. The insulin contents tended to be stable within 3–5 days. The serum insulin contents insulin and blank control groups remained in equilibrium at a specific concentration. The trends for the α-MSH-containing groups were similar to that of the insulin-containing groups, but the initial α-MSH contents were higher and the significance for the GM4-α-MSH group was weaker than the α-MSH group (Figure 5(B)).

From mouse tissue distribution, Figure 5(C) shows that the distribution of the recombinant strain GM4-ΔTS-PGK1-CCT carrying insulin in the liver organs was as follows: liver, kidney, heart, lung and spleen, and reached the highest level at 2 h, especially in the liver, where its concentration reached 5.36 pg·g^−1^. This may be mainly related to lipid metabolism in the liver. We suspect that insulin encapsulated in lipid droplets is likely to enter the lymphatic system along with chylomicron lipid formation and finally enter the bloodstream for transportation into various tissues of the body. Analysis of the distribution of α-MSH in tissues revealed that GM4-ΔTS-PGK1-CCT carrying α-MSH significantly increased its distribution in liver, lung, spleen, and kidney compared with the GM4-α-MSH and α-MSH groups, especially in the liver.

### 3.10. Study on the GM4-ΔTS-PGK1-CCT strain carrying insulin in type II diabetic mice

After 72 hours of modeling, no death was found in the mice, and there were no significant differences in body indexes, such as diet and body weight, from before modeling. After modeling, the mice showed decreased activity and increased water intake and urine volumes.

As shown in Figure 5(E), type II diabetic mice that were intragastrically administered PBS containing insulin did not show significant changes in their blood glucose, which may be caused by insulin inactivation by various enzymes in the gastrointestinal tract. However, after subcutaneous injection of insulin-containing PBS solution, the blood glucose concentration in mice maintained low levels within 2 hours after injection. The reduction in the blood glucose caused by the direct injection of insulin is due to its short half-life; this means that lower blood sugar levels can be maintained for only a relatively short period of time. After treatment with the recombinant GM4-ΔTS-PGK1-CCT-insulin strain by gavage, the blood glucose levels of the mice changed significantly within 1–4 h and remained at a relatively low level after 6 h. The ability of the wild GM4 strain to lower blood glucose was weaker than the recombinant strain carrying insulin. After treatment with the recombinant GM4-ΔTS-PGK1-CCT-insulin strain and GM4-insulin strain by gavage, the strains entered the body, were not completely destroyed, and effectively lowered blood glucose levels. Therefore, the recombinant GM4-ΔTS-PGK1-CCT-insulin strain can be used as a producer of exogenous insulin for the treatment of diabetes, and has a significant hypoglycemic effect.

## 4. Discussion

The CCT enzyme is not only the rate-limiting enzyme in the lecithin synthesis pathway, but also a key enzyme affecting lipid synthesis and accumulation *in vivo*. To make the oil in our high-yielding oil *R. glutinis* GM4 strain from the early isolated candidate oleaginous microorganisms more suitable for drug liposomes, we constructed a TS-deficient strain GM4-ΔTS by homologous recombination and then transferred the rate-limiting enzyme CCT from lecithin synthesis into the GM4-ΔTS strain. The exogenous CCT gene was highly expressed in the GM4-ΔTS strain, and the lecithin contents in the recombinant GM4-ΔTS-PGK1-CCT strain increased from 40.8% to 60.7%, while the intracellular lipid droplets became smaller and increased. This result also confirmed the key role of the CCT enzyme in the phospholipid synthesis pathway. As shown in Figure 5(F), the high expression of the CCT gene can not only increase phospholipid anabolism but also increase the synthesis and accumulation of PUFAs such as phosphatidylcholine and phosphatidylserine in cells, thereby increasing the tolerance of nonpolar peptides or other lipophilic small molecule drugs.

In previous studies, our laboratory determined that H22-LP (the conservative sequence is NAHCALL) is an antagonist peptide for the broad-spectrum chemokine receptor US28, which has been extensively studied (Lu et al., 2010; Liu et al., 2012; Shi et al., 2014); for example, it inhibited the effect of human cytomegalovirus by directly interacting with virus particles. MSH is a neuroimmunomodulatory peptide derived from pro-opiomelanocortin (POMC). It consists of 13 amino acids and its amino acid sequence is SYSMEHFRWGKPV (Catania et al., 2010; Gupta & Wish, 2018). It can be produced by converting enzymes in mice. Insulin is a protein hormone secreted by islet β-cells in the body and its amino acid sequence is GIVEECCASVCSLYELEDYCD-FVDEHLCGSHLVEALYLVCGERGFFYTPKA. In this study, we successfully introduced exogenous polypeptides (Aliphatic index: H22-LP > insulin > α-MSH and GRAVY: H22-LP > insulin > α-MSH (according to Figure 4 (D,E,F)) into cells using recombinant strains with high expression of CCT; however, the specific reasons for the entrance of these exogenous polypeptides into the cells requires further study. It is clear that the lipophilic properties of the peptides promote the entry of the drugs into the cells. Therefore, it is possible to use this liposome as the oral drug delivery system for other lipophilic compounds.

Next, to study the oral effects of the modified live-cell liposomes, different levels of insulin were detected in serum and tissues after oral administration of the recombinant GM4-ΔTS-PGK1-CCT-insulin strain, showing the delivery of insulin in mice. Moreover, the oral administration of the insulin-bearing recombinant GM4-ΔTS-PGK1-CCT-insulin strain in type II diabetes mellitus mice significantly reduced blood glucose levels *in vivo*, indicating that the recombinant strain we constructed successfully carries the exogenous polypeptide into the body. Therefore, our experimental results show that the small lipid droplet cells modified by the CCT enzyme are well-tolerated for the insulin, H22-LP, and α-MSH polypeptide drugs, and that the peptide drugs can be orally carried into the body in the form of bioactive bacteria capsules. Compared with traditional peptide drugs, these live-cell capsules have strong stability *in vivo*, a prolonged half-life (Tang et al., 2004), good bioavailability, etc. They also display a sustained release effect, avoiding the corresponding weakness associated with naked peptide applications, and further expanding the applications for many small molecular peptide drugs.

At present, polypeptides are known for their low molecular weight and being directly absorbed by humans, which has recently been paid more and more attention. Oral bioactive bacteria capsules containing peptide drugs also highlight its significance in many respects. Protein and peptide drugs are widely distributed in nature and play an important role in the prevention and treatment of diseases. Clinically, many peptide drugs have excellent efficacy, such as erythropoietin (EPO) (Bertolini et al., 2009), granulocyte-macrophage colony stimulating factor (GM-CSF), and interleukin-2 (IL-2), which are mainly used to stimulate hematopoiesis, antitumor effect and other aspects (Moghaddam et al., 2018). For example, insulin can be hydrolyzed and inactivated by proteolytic enzymes in the stomach and duodenum. Our study showed better oral bioavailability for *R. glutinis* bioactive cell capsules introduced with insulin. Thus, we report that *R. glutinis* bioactive bacteria ‘capsule’ system may become a new theoretical and application breakthrough for the drug liposomes.

There is also a possibility of oral administration of insulin using protective measures. It has been reported that insulin molecules can be absorbed into the blood through the intestinal wall. The absorption rate of the small intestine is the highest, though the absorption rate of colon and rectum is also high (Cullis, 1976). It has been confirmed that insulin molecules enter the intestinal environment through transport via intestinal epithelial cells and then penetrate into the plasma membrane of capillary endothelial cells to enter the blood circulation (Storm et al., 1987). Scientists (Defrise-Quertain et al., 1980) used an azopolymer that can be degraded by microbes in the colon to coat insulin pellets and added salicylic acid as an absorption enhancer. The pellets could effectively avoid enzyme damage in the stomach and small intestine after oral administration, followed by smooth entrance into the upper part of the colon, degradation of the coating layer, release and absorption of the drug to produce hypoglycemic effects. It is clear that our *R. glutinis* insulin bioactive bacteria capsules can resolve the intestinal epithelial absorption, but the intestinal absorption mechanism needs to be further studied.

Antibiotics such as erythromycin, ampicillin, chloramphenicol, and other resistance genes are often used as markers for cloning and screening, but plasmid vectors with antibiotic resistance as the selection pressure have many safety problems. For example, if a patient ingests micro-ecological preparations with antibiotics, the resistance genes may transmit and spread through the bacteria. Therefore, it is particularly important to construct a carrier system with non-antibiotic resistance as the selection pressure. In this study, in the gene cloning experiment, we used a housekeeping gene encoding TS in *R. glutinis*, which is an enzyme required for DNA synthesis, that participates in catalyzing the methylation of uracil deoxynucleotides through 5,10-dimethyltetrahydrofolate to synthesize 5′-thymidine deoxynucleotides. When TS is mutated or knocked out, cells cannot grow without thymidylate or thymine, unless it is transformed by a functional gene. Therefore, we constructed a TS-deficient strain as recipient bacterium and cloned a homologous intact receptor-deficient gene into a nonresistant plasmid. As a selection pressure for the stable expression of the exogenous gene, it can not only be used to quickly identify and isolate the transformed strains, but also plays a screening role for positive clones. This way the lipid can be synthesized normally *in vitro* and the exogenous peptides can be successfully introduced when a specific intracellular lipid droplet accumulation is reached. At this point in time, the auxotrophic medium is withdrawn and the transformed drug-loaded carrier bacteria are relatively stopped from being metabolized. Thus, the stability of the drug-loaded cells is better preserved, and a safe and controllable drug-loading system is truly provided. At the same time, this study also proved that recombinant *R. glutinis* strains can proliferate in special medium (including TS) due to the presence of defective TS, but can not proliferate *in vivo* and *vitro* in the absence of TS, making the strain a safe, controllable release agent for *in vivo* drugs.

Liposomes generally undergo rapid systemic clearance due to their uptake by the mononuclear phagocytic system (Haran et al., 1993; Cabanes et al., 1998), and several engineering strategies have been applied to improve their *in vivo* performance. These strategies include the attachment of site-directed surface ligands, such as antibodies (immune liposomes) (Modi et al., 2012; Allen & Cullis, 2013), positive charges (cationic liposomes) (Charrois & Allen, 2004; Laginha et al., 2005), and peptides (liposomes targeting peptides) (Kim et al., 2001; Ran et al., 2018), or using physiological conditions such as temperature or pH changes inherent to the target tissue to produce stimulating reactive liposomes such as thermosensitive liposomes (Ando et al., 2015; Burke et al., 2017) and pH-sensitive liposomes (Essam et al., 2015; Kanamala et al., 2018), respectively. However, the oral living cell liposomes in this study have unique application advantages, such as protecting the polypeptide drug the gastric acid in the digestive tract, providing a good protective barrier for non-biological polar molecules, and the natural sustained release effect for oral administration. However, using yeast as a living cell liposome to encapsulate other molecular drugs as well as peptide drug molecules requires further research.

## 5. Conclusion

In this paper, the *R. glutinis* recombinant strain GM4-ΔTS-PGK1-CCT overexpressing the exogenous CCT gene was successfully realized. This recombinant strain with high CCT expression could carry insulin, H22-LP, and α-MSH into the cells; additionally, the strain no longer proliferated *in vivo* and was a safe and controllable vehicle for polypeptide drug delivery. After oral administration of the strain carrying the polypeptide, the corresponding fatty acid and lecithin contents in mice were significantly increased. Similar effects were observed in the type 2 diabetic mice, indicating that the recombinant strain GM4-ΔTS-PGK1-CCT can be used as a living cell liposome to delivery polypeptide drugs, as this was the aim in this experiment. Among them, the constructed recombinant strain GM4-ΔTS-PGK1-CCT served as a living cell liposome to delivery polypeptide drugs *in vitro*. After oral administration, the recombinant strain cells no longer proliferate; thus, it is safe and controllable *in vivo*. The feasibility and preference of the recombinant strain GM4-ΔTS-PGK1-CCT carrying insulin, H22-LP, and α-MSH were also discussed.

The results of this study report for the first time, establish the feasibility of using *R. glutinis* as a living cell liposome for the oral delivery of polypeptide drugs. As a bioactive bacterium capsule, it is expected to be used in the delivery systems and as a new administration route for many polar small molecular drugs including drug peptides and compounds.

## Supplementary Material

Supplementary_Information.rar
